# From Batch to the Semi-Continuous Flow Hydrogenation of *p*NB, *p*NZ-Protected Meropenem

**DOI:** 10.3390/pharmaceutics15051322

**Published:** 2023-04-23

**Authors:** Marziale Comito, Riccardo Monguzzi, Silvia Tagliapietra, Angelo Maspero, Giovanni Palmisano, Giancarlo Cravotto

**Affiliations:** 1 Dipartimento di Scienza e Tecnologia del Farmaco, University of Turin, Via Pietro Giuria 9, 10125 Turin, Italy; marziale.comito@unito.it (M.C.); silvia.tagliapietra@unito.it (S.T.); 2 Research and Development, ACS Dobfar SpA, Via Paullo 9, 20067 Tribiano, Italy; riccardo.monguzzi@acsdobfar.it; 3 Dipartimento di Scienza e Alta Tecnologia, University of Insubria, Via Valleggio 9, 22100 Como, Italy; angelo.maspero@uninsubria.it (A.M.); giovanni.palmisano@uninsubria.it (G.P.)

**Keywords:** flow chemistry, microwave-assisted, semi-continuous synthesis, meropenem, hydrogenation, drug synthesis, heterogeneous catalysis, miniaturization, sustainability

## Abstract

Meropenem is currently the most common carbapenem in clinical applications. Industrially, the final synthetic step is characterized by a heterogeneous catalytic hydrogenation in batch mode with hydrogen and Pd/C. The required high-quality standard is very difficult to meet and specific conditions are required to remove both protecting groups [i.e., *p*-nitrobenzyl (pNB) and *p*-nitrobenzyloxycarbonyl (pNZ)] simultaneously. The three-phase gas–liquid–solid system makes this step difficult and unsafe. The introduction of new technologies for small-molecule synthesis in recent years has opened up new landscapes in process chemistry. In this context, we have investigated meropenem hydrogenolysis using microwave (MW)-assisted flow chemistry for use as a new technology with industrial prospects. The reaction parameters (catalyst amount, T, P, residence time, flow rate) in the move from the batch process to semi-continuous flow were investigated under mild conditions to determine their influence on the reaction rate. The optimization of the residence time (840 s) and the number of cycles (4) allowed us to develop a novel protocol that halves the reaction time compared to batch production (14 min vs. 30 min) while maintaining the same product quality. The increase in productivity using this semi-continuous flow technique compensates for the slightly lower yield (70% vs. 74%) obtained in batch mode.

## 1. Introduction

Carbapenems are currently considered to be lifesaving drugs [1], with their broad-spectrum activity meaning that they play a vital role for human health. They have the greatest potency of all β-lactam antibiotics against Gram-positive and Gram-negative bacteria. Their medical use is so crucial and essential that they have been defined “last-line antibiotics” or “antibiotics of last resort”, as they are the most effective weapon against known and suspected multidrug-resistant (MDR) bacterial infections [2,3,4,5,6,7,8,9,10,11,12,13,14,15,16,17,18,19,20,21,22,23,24,25,26].

**Figure 1:**
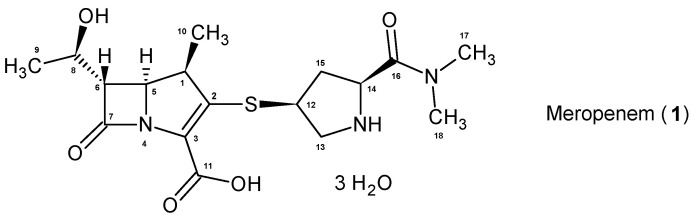
Meropenem (**1**).

Part of the carbapenem antibiotics subgroup, meropenem (**1,** Figure 1) is currently the most widely used in clinical treatment [27,28,29]. Its worldwide market was valued at USD 1.7 billion in 2021 and is expected to grow to USD 2.1 billion by 2027, making this drug a blockbuster [30]. Alone, it made up 43% of the USD 3.9 billion in global sales of the carbapenem family in 2021 [31]. It was discovered by Sumitomo Dainippon Pharma, now Sumitomo Pharma, in 1983 [32,33,34], and was approved by the Food and Drug Administration (FDA) in 1996 [35]. The drug is sold under the brand name Merrem™, as a generic drug substance, or in combination with vaborbactam, part of a new generation of β-lactamase inhibitors, under the brand name Vabomere™. It is administered intravenously in both forms, and is listed on the World Health Organization’s list of Essential Medicines thanks to its importance [36,37,38,39,40,41,42,43,44,45,46].

**Scheme 1:**
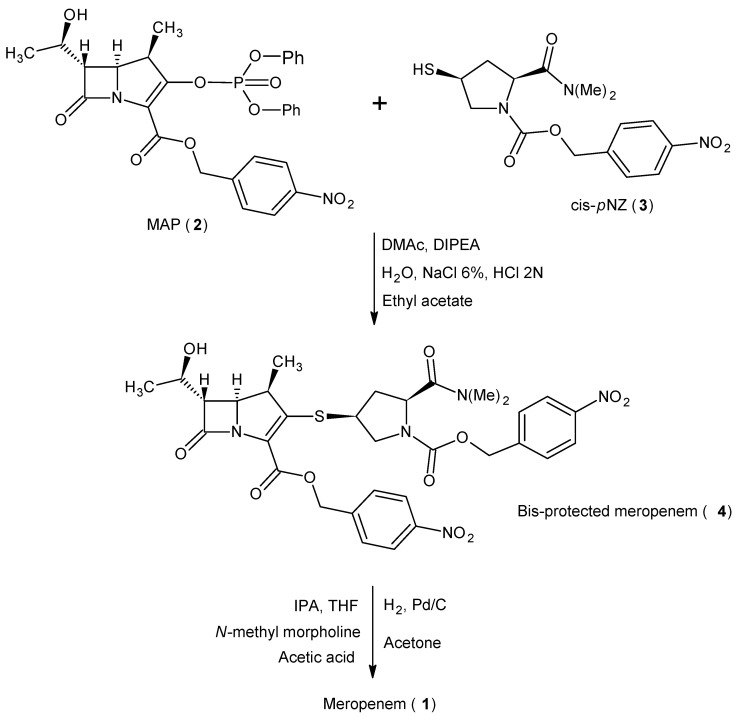
Meropenem (**1**) synthesis from MAP (**2**) and cis-*p*NZ (**3**).

**Figure 2:**
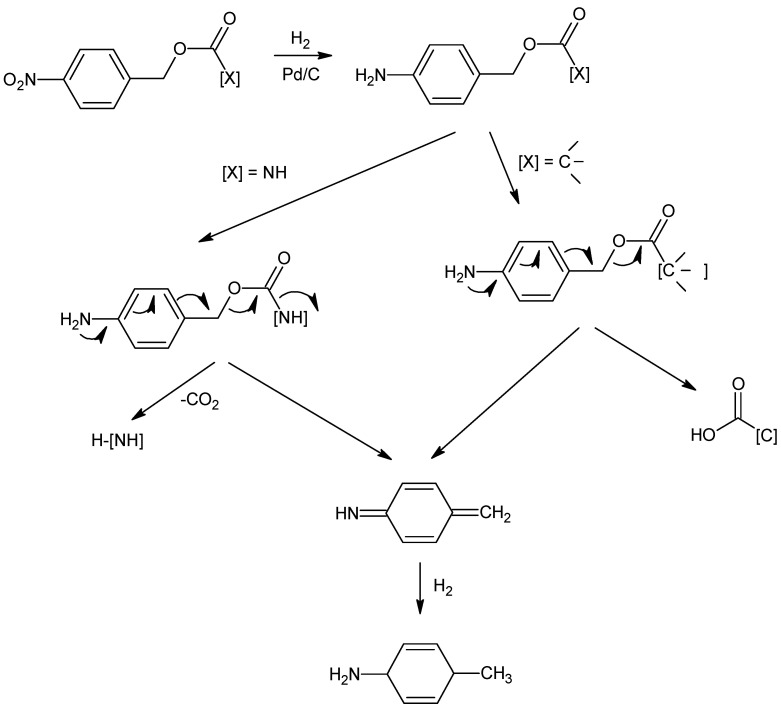
Reduction mechanism of *p*NB/*p*NZ protecting groups.

The literature reports different synthetic routes for its preparation [32,33,34,47,48,49,50,51,52,53,54,55,56,57,58,59,60,61,62,63,64,65,66,67,68]. The most widely used industrial synthesis includes more than twenty steps characterized by protection with *p*-nitrobenzyl (*p*NB) and *p*-nitrobenzyloxycarbonyl (*p*NZ) on the carbapenem scaffold. This strategy, which allows the preparation of the drug with the correct stereochemical configuration (β-methyl in position 1), is still considered the most efficient and competitive in the world market. The high stability conferred to the β-lactam core during all chemical steps by these protecting groups with a high steric hindrance has been the main advantage of their use for more than 30 years. As depicted in Scheme 1, the condensation between 4-nitrobenzyl(4*R*,5*S*,6*S*)-3-[(diphenylphosphono)-oxy]-6-[(1*R*)-1-hydroxyethyl]-4-methyl-7-oxo-1-azabicyclo[3,2,0]hept-2-ene-2-carboxylate (**2**, MAP) and (2*S*,4*S*)-2-dimethylaminocarbonyl-4-mercapto-1-(4-nitrobenzyloxycarbonyl)pyrrolidine (**3**, cis-*p*NZ) gives bis-protected meropenem (**4**). Batch-mode hydrogenation with palladium over carbon (Pd/C) under pressure simultaneously removes the *p*-nitrobenzyl (*p*NB) and *p*-nitrobenzyloxycarbonyl (*p*NZ) protecting groups, according to mechanism reported in Figure 2. In the first step, the nitro group is reduced to the *p*-aminobenzyl(oxycarbonyl) intermediate, which spontaneously collapses to the quinonimine methide and the deprotected compound by 1,6-electron-pair shift [69]. The subtle equilibrium between the hydrogenation operating parameters and the stability of the carbapenem skeleton has always been the key to the success of this reaction, leading all manufacturers to balance these two factors. The catalytic system was characterized by the presence of *N*-methylmorpholine and acetic acid as buffers, which allowed it to operate at pH = 6.5–6.8 throughout the reaction time, ensuring the stability of both reagent and product. Water and isopropyl alcohol met the requirements of green chemistry and sustainability, and the waste was easy to handle. Catalyst filtration, workup, and subsequent first crystallization provides crude meropenem (1). The product is purified by a second crystallization to reach the quality standard before it is sterilized and becomes an injectable drug. Hydrogenation reactions pose significant risks for a production plant, and this can only be mitigated in part by heavy investment. The implementation of batch-mode manufacturing requires a bunker facility, and the engineered architecture must be designed to restrict uncontrolled events and any possible damage must be contained. The risk to people and the surrounding area has to be under control [70,71,72,73,74]. The considerable manufacturing capacity needed to support massive global demand, the operational accuracy needed to obtain a high-grade product and the high flammability of Pd/C are all real and routine issues that must be tackled in the commercial production of meropenem (**1**). Batch production is no longer adequate in the industrial manufacturing of pharmaceuticals, where a balance between streamlining and increased productivity is required to compete in the global active pharmaceutical ingredients (APIs) market. In addition, the high-quality standard required is very challenging to satisfy due to the instability of the carbapenem skeleton during the hydrogenation step.

In the context of the current drive towards the adoption of emerging technologies for the synthesis of APIs, functional intermediates and natural compounds, we have investigated the hydrogenation of bis-protected meropenem (**4**) under microwave (MW)-assisted flow chemistry. We have chosen this technology because of its well-known benefits to mass and heat transfer, miniaturization, safety, speed and sustainability. Semi and continuous flow chemistry have traditionally been used and developed as a safer and more efficient method when batch operations are deemed to be too dangerous. The small operational units minimize exposure to hazardous reagents and permit safe handling. The integration and modularization of operations allow for much easier study and production scale-up. Automated monitoring techniques and the reduction in the probability of human error are significant industrial advantages, making this technology flexible and agile. Although flow chemistry does not entirely eliminate safety concerns in industry, it does decrease risk factors to levels at which they are easier to manage. Furthermore, the use of MW irradiation makes this a highly efficient and green technology that can fulfill energy-saving and sustainability requirements. Faster heating rates and uniform heat distribution mean that MW chemistry is one of the most attractive of the emerging technologies [75,76,77,78,79,80,81,82,83,84,85,86].

MW-assisted flow chemistry has been widely applied over the last two decades to synthesize myriad organic compounds, demonstrating its versatility and potential. From unsaturated organic substrates to heterocycle synthesis and metal-catalyzed chemistry, this technology has played a central role in the development of sustainable multigram preparations that meet the dual requirement of process intensification and reduced production costs. It has proven to be a promising hybrid technique that can link lab-scale investigations and pilot processes, and thus meet industrial challenges [87,88,89,90,91,92,93,94]. In addition, the FDA and the European Medicines Agency (EMA) have strongly encouraged the study and implementation of this innovative, sustainable and environmentally friendly technology in API synthesis [95,96]. β-lactam chemistry has remained anchored to developments from many years ago and has only recently achieved renewal in a few cases [97,98]. With this revolution in place, MW-assisted flow chemistry could really upend acquired certainties in the near future.

The present work demonstrates the effective implementation of a new emerging technology in the synthesis of a blockbuster drug. It describes a new attractive protocol that is suitable for industrial application as an alternative to batch mode. MW-assisted flow chemistry fulfills the required high-quality standard, as hydrogenation is the final step in meropenem (**1**) manufacture. The quality product is comparable to commercial drug. The parameters have been investigated with an eye to process intensification and further scale-up. The carbapenem skeleton stability has been preserved by working under mild conditions and by exploiting its intrinsic characteristics. We believe that this work may be pioneering in API synthesis and indispensable to the development of this technology in the pharmaceutical panorama.

## 2. Materials and Methods

### 2.1. Chemistry

MAP (**2**), cis-*p*NZ (**3**), 10% Pd/C and the solvents were kindly provided by ACS Dobfar. All other reagents were purchased from Merck KGaA (Darmstadt, Germany).

The Agilent 1200 series HPLC system, NMR (Bruker 400, Munich, Germany), and Karl Fischer titration were the analytical instruments used to identify and analyze the products.

#### 2.1.1. General Procedure for the Synthesis of (4*R*,5*S*,6*S*)-(*p*-Nitrobenzyl)-3-[[(3*S*,5*S*)-1-(*p*-nitrobenzyloxycarbonyl)-5-(dimethylaminocarbonyl)-3-pyrrolidinyl]thio]-6-[(1*R*)-1-hydroxyethyl]-4-methyl-7-oxo-1-azabicyclo[3.2.0]hept-2-ene-2-carboxylate (**4**)

MAP (**2**, 94.0 g, 158.0 mmol) and cis-*p*NZ (**3**, 59.2 g, 167.6 mmol) were dissolved in 400 mL of dimethylacetamide and cooled to −10 °C. Over the course of 15 min, diisopropylethylamine (DIPEA) (88 mL, 505.3 mmol) was added dropwise at a temperature not exceeding −7 °C. The mixture was stirred for 90 min at −10 °C. An amount of 1200 mL of pre-cooled (0–5 °C) ethyl acetate was then added over 30 min with the temperature being maintained at −10 °C, and finally 800 mL of ice water was added, which raised the temperature to 0–5 °C. The aqueous phase was separated and extracted with 600 mL ethyl acetate at 0–5 °C. The combined organic phase was extracted twice, each time with a cold mixture of 320 mL 6% aqueous NaCl solution and 80 mL 2N hydrochloric acid. Finally, the organic phase was extracted with 400 mL phosphate buffer solution pH 7.0. The organic phase was separated, filtered to clarify, and the filter then washed with 40 mL of ethyl acetate. The filtrate was concentrated to 360 g at 40 °C and made up to 440 g with fresh ethyl acetate. The solution was stirred for 18 h at 20 °C in order to crystallize the crude bis-protected meropenem (**4**). In order to complete the crystallization, 140 mL of heptane were added dropwise, and the slurry was stirred for a further 30 min. The product was isolated by filtration, washed twice, each time with 200 mL of heptane, and dried for 16 h at 40 °C under vacuum to give the crude title compound (**4**) (101.2 g, 91.8% yield) as an off-white solid. The HPLC assay gave a result of 98.3%. *Purification:* crude bis-protected meropenem (**4**, 100 g, 143.3 mmol) was dissolved in 1680 mL ethyl acetate. The slurry was stirred at 20 °C for 90 min. An amount of 140 mL cyclohexane was added in order to complete the crystallization and the slurry was stirred for 2.5 h. The pure bis-protected meropenem (**4**) was isolated via filtration, washed with 200 mL cyclohexane, and dried for 16 h at 40 °C under vacuum to give the pure title compound (**4**) as a white solid (91.1 g, 91.1% yield). The HPLC assay gave a result of 99.6%.

Signals in the ^1^H- and ^13^C-NMR spectra are split due to the presence of *p*NZ rotamers in a ~56:44 ratio at 300 K.

^1^H NMR: (400 MHz, DMSO-d_6_, 300 K): δ_H_ 8.26, 8.25 (4H, 2 × d, ^3^J 8 Hz); 7.76 (2H, d, ^3^J 8 Hz); 7.68, 7.56 (2H, 2 × d, ^3^J 8 Hz); 5.50 & 5.34 (2H, AB syst, ^2^J 14 Hz); 5.30–5.05 (3H, m); 4.88, 4.80 (1H, 2 × t, ^3^J 8 Hz); 4.35–4.30 (1H, m); 4.28, 4.18 (1H, 2 × dd, ^3^J 10 Hz, ^3^ J 7 Hz); 4.10–4.00 (1H, m); 3.96–3.80 (1H, m); 3.70–3.60 (1H, m); 3.37–3.32 (1H, m); 3.28, 3.21 (1H, 2 × t, ^3^J 10 Hz); 3.07, 3.01 (3H, 2 × s); 2.98–2.80 (4H, m); 1.75–1.60 (1H, m); 1.21 (3H, d, ^3^J 7.5 Hz); 1.19 (3H, d, ^3^J 7.5 Hz), Figure S1. Underlined peaks are ascribed to the major rotamer.

^13^C NMR: (100 MHz, DMSO-d_6_, 300 K): δ_C_ 175.02 (C), 174.90 (C), 171.51 (C), 171.15 (C), 160.73 (C), 153.75 (C), 153.48 (C), 151.15 (C), 151.08 (C), 147.86 (C), 147.72 (C), 145.46 (C), 145.50 (C), 145.46 (C), 145.50 (C), 129.08 (CH), 128.82 (CH), 128.56 (CH), 124.58 (C), 124.45 (C), 124.27 (CH), 124.18 (CH), 65.73 (CH_2_), 65.63 (CH_2_), 64.94 (CH), 64.89 (CH), 57.03 (CH), 56.67 (CH), 56.15 (CH), 43.83 (CH), 40.04 (CH), 37.15 (CH), 37.07 (CH), 36.54 (CH_2_), 35.82 (CH_2_), 22.51 (CH_3_), 22.46 (CH_3_), 17.84 (CH_3_), Figure S2.

#### 2.1.2. General Procedure Applied in All Batch-Mode and MW-Assisted Flow-Mode Experiments for the Synthesis of Meropenem (**1**)

Bis-protected meropenem (**4**, 28.5 g, 40.8 mmol), acetic acid (1.4 g, 23.3 mmol), *N*-methyl morpholine (4.2 g, 41.5 mmol), 257 g tetrahydrofuran (THF), 282 g isopropyl alcohol and 428 g of water made up the starting solution, which was kept constant across all of the experiments. The initial concentration was about 40 mmol/L. *N*-methyl morpholine and acetic acid were used as buffers during the reaction, allowing it to work at pH = 6.5–6.8. The catalyst 10% Pd/C was set as indicated in Table 1. The total volume of slurry during hydrogenation was approximately 1000 mL, and had a density of 0.94 g/mL. After the hydrogenation experiments, the catalyst was filtered and the aqueous phase was extracted with 285 mL of dichloromethane at room temperature (solution pH = 5.3–5.5). An amount of 2850 mL of acetone was added to the aqueous phase for 3 h at 0–5 °C to crystallize the meropenem (**1**). The slurry was stirred for 60 min at 0–5 °C, filtered and washed with 30 mL of acetone. The crude product was dried for 30 min under vacuum with nitrogen flow. The yield values are listed in Table 1 and Table 4. The HPLC assay gave a result of 98%. *Purification:* crude meropenem (**1**, 5.0 g, 11.4 mmol) was dissolved in 150 mL water with NaHCO_3_ (0.1 g, 1.2 mmol) at 35 °C. After 5 min, the solution was cooled to 0–5 °C and treated with norite charcoal (0.5 g) for 1 h. The charcoal was removed by filtration and the filter cake was washed with 5 mL water. An amount of 0.5 g of seed was added, and the solution was stirred for 1 h at 0–5 °C. An amount of 450 mL acetone was added dropwise within 4 h and stirred for a further 30 min. The solid was collected by filtration, washed with 10 mL acetone and dried under vacuum for 1 h at room temperature to give pure meropenem (**1**) as a white solid (4.2 g, 84% yield). The HPLC assay gave a result of 99.5%.

^1^H NMR (400 MHz, D_2_O): δ_H_ 1.15 (3H, d, ^3^J 7.6 Hz, 10-Me), 1.23 (3H, d, ^3^J 6.4 Hz, 9-Me), 1.91 (1H, appt quint, J 6.8 Hz, H-15α), 2.94^§^ (3H, s, 17-Me), 3.02^§^ (3H, s, 18-Me), 3.06 (1H, m, H-15β), 3.31 (1H, dq,^2^ J 14.8 Hz, ^3^J 7.2 Hz, H-1), 3.4–3.5 (2H, m, H-6/H-13α), 3.71 (1H, dd, ^2^J 12.4 Hz, ^3^J 6.4 Hz, H-13β), 4.00 (1H, appt quint, J 6.4 Hz, H-12), 4.15–4.25 (2H, m, H-5/H-8), 4.75 (1H, t, ^3^J 8.8 Hz, H-14), Figure S3.

^13^ C NMR (100 MHz, D_2_O): δ_C_ 176.75 (C-11), 168.0 (C-16), 167.75 (C-7), 137.6 (C-2), 134.0 (C-3), 65.3 (C-8), 59.0 (C-6), 58.4 (C-14), 56.1 (C-5), 52.35 (C-13), 42.65 (C-1), 40.6 (C-12), 36.8 (C-18)^#^, 36.0 (C-17)^#^, 20.9 (C-9), 16.0 (C-10), Figure S4.

^§#^ may be interchanged, appt: apparent (first-order approximation).

### 2.2. Hydrogenation Reactions

#### 2.2.1. Reaction in Batch Mode

**Scheme 2:**
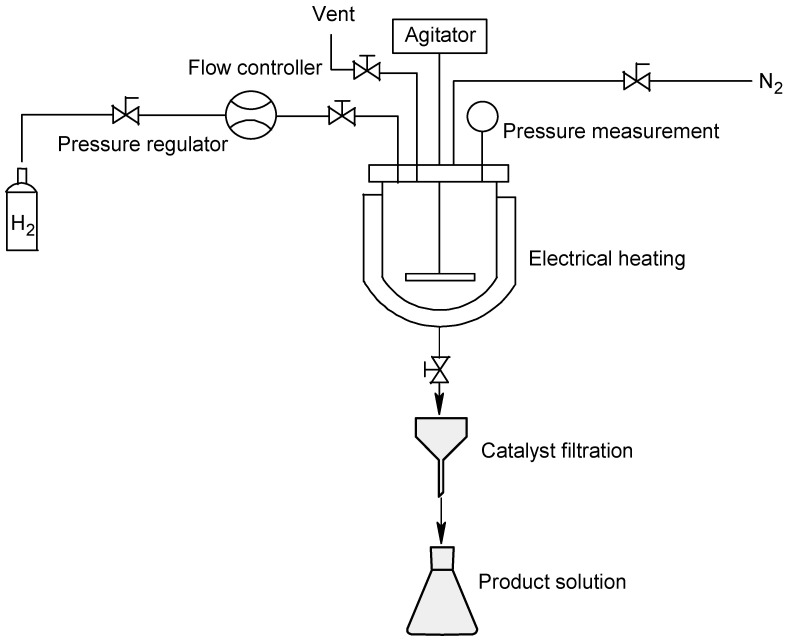
Batch-reactor configuration.

A Parr reactor series 4578 High Pressure/High Temperature (Parr Instrument Company, Moline, IL, USA) was used for the batch-mode experiments. A floor stand with a pneumatic lift mounted the fixed-head reactor, which has a volume of 1800 mL and is capable of working at up to 345 bar pressure and 500 °C temperature. A mass flowmeter placed in the hydrogen line allowed the reactor pressure, which was measured with a pressure measurement on the top of the reactor, to be controlled. The temperature was kept constant using an electrically driven serpentine. An impeller performed slurry stirring correctly. Scheme 2 depicts the reactor configuration used for batch-mode hydrogenation.

The bis-protected meropenem (**4**) solution was introduced into the reactor with the catalyst. Inertization was performed with three nitrogen purges at 5 bar, and the reactor was subsequently purged three times with hydrogen at 5 bar. The stirrer was set and the hydrogen valve opened. A pressure ramp from 0 bar to final pressure (3 bar/min) was applied, as was a temperature ramp (2.5 °C/min). The temperature was kept constant at the final reaction temperature, and the pressure was maintained using the hydrogen flowmeter (Table 1). The reaction was run until hydrogen uptake was completed, at which point the hydrogen flow was interrupted and the solution cooled to room temperature. The reactor was slowly vented to release the hydrogen and underwent inertization with three nitrogen purges at 5 bar. The slurry was filtered using nitrogen pressure on a Buchner funnel to remove the catalyst, allowing solution analysis to then be performed.

#### 2.2.2. Reaction in MW-Assisted Flow Mode

**Scheme 3:**
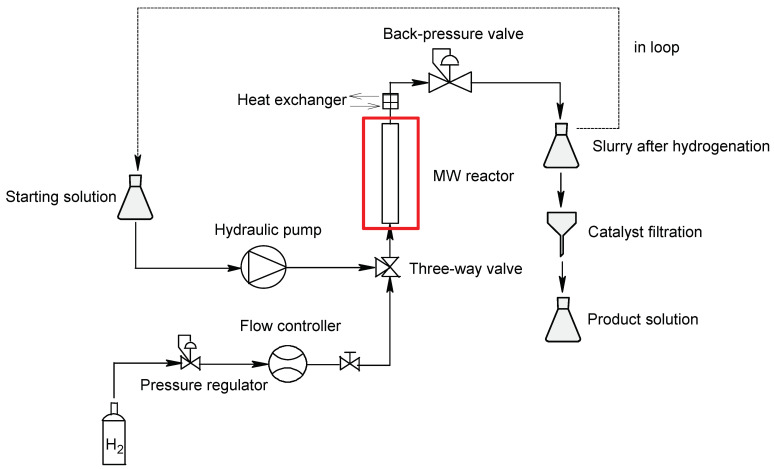
FlowSYNTH reactor configuration.

MW-assisted flow hydrogenation was carried out in the FlowSYNTH reactor (Milestone Srl, Bergamo, Italy), which is a multimode system that operates at 2.45 GHz and is equipped with a vertical PTFE-TFM flow-through reactor that can work up to a maximum temperature of 200 °C and 20 bar of pressure, in open or closed loop modes. A three-way connection fitted with a non-return valve allows the starting solution and hydrogen feeds to be pumped simultaneously. A back-pressure valve placed on the top of the reactor, after a water-cooled heat exchanger, ensures pressure control and flow-stream decompression, allowing the solution to be collected. For a single cycle run, the slurry was run in the reactor (V*_R_* = 200 mL) using a hydraulic pump and was collected in an Erlenmeyer flask after catalyst filtration, and this was performed in loop for multiple-cycle runs. The starting solution and hydrogen flowed from the bottom to the top of the reactor, with the applied MW power being modified in real time to maintain the predefined temperature. The pressure was kept constant using a hydrogen mass flowmeter at the indicated pressure. Integrated reactor sensors continuously monitored the internal pressure, temperature and applied power inside the reactor for each run. Leak tests were successfully performed first with water and then with isopropyl alcohol and nitrogen to test the pressure control. A general scheme of the FlowSYNTH reactor is depicted in Scheme 3.

## 3. Results and Discussion

As a first step, we focused on investigating the preferred reaction parameters for batch-mode hydrogenation in order to subsequently upload them for flow-mode use. The key factors in determining successful *p*NB/*p*NZ removal are pressure, temperature and Pd/C amount. As reported in Table 1, catalyst load has a decisive impact on the meropenem (**1**) yield from hydrogenation, and influences how the process is run. Upon performing the deblock in the Parr reactor at 20 bar and 37 °C, we observed a drop in yield and an increase in the reaction time when the catalyst amount per test was halved. The same trend was observed when working at lower pressure (6.8 bar) and temperature (30 °C), but starting with a greater catalyst load. An inversely proportional relationship exists between these three parameters, and this had a significant impact on the choice of reaction conditions for the subsequent flow-mode experiments. In industry, reducing the amounts of the highly expensive catalysts would undoubtedly be impactful, but incompatible with the instability of the carbapenem nucleus under harsh conditions. We observed a reduction in yield even when the dry catalyst percentage and pressure value were kept constant, but the temperature increased (35 °C and 45 °C), demonstrating once again the need to work under moderate conditions. In all tests, the reagent conversion was complete, meaning that the reaction generated different undetectable impurities. Using a catalyst amount of 21% dry w/w for bis-protected meropenem (**4**) at 30 °C and 6.8 bar granted a meropenem (**1**) yield in solution, after catalyst filtration, of 89%. We decided to only crystallize the hydrogenated solution formed under moderate conditions to have a comparison with the products investigated in flow mode.

**Figure 3:**
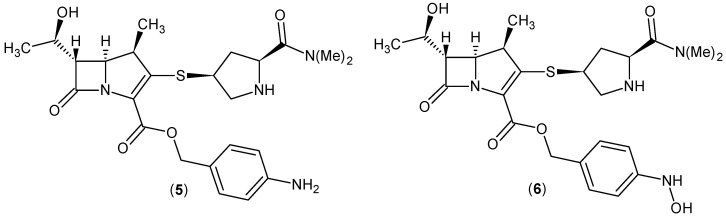
Impurities in crude meropenem (**1**), from Entry 4, investigated using LC-MS.

The work-up of the aqueous solution together with the purifying effect of acetone, used in crystallization, led to the isolation of crude meropenem (**1**) in Table 1 with an assay result of 98% and a yield of 74%. Compared to the data reported in literature [48], our system has proven to perform better. An LC-MS analysis (Supplementary Figure S5) of the product derived from Entry 4 identified two impurities, *p*-amino (**5**, 0.7%) and *p*-hydroxylamino (**6**, 0.3%) derivatives, which arose after the removal of the *p*NZ side chain from the stepwise reduction in the nitro group in the transient mono-protected *p*NB compound (Figure 3). These structures would seem to indicate the reaction mechanism proposed in Figure 2. The purification of the crude product purged these impurities and gave a pure product with 99.5% assay result, comparable to commercial product.

Meropenem (**1**) hydrogenation under MW-assisted flow chemistry has never been investigated. Moreover, this technology appears to us to be the most suitable for a study into the potential industrial production of Meropenem (**1**) as the drug is a blockbuster and manufactured on a large scale.

Having identified the best conditions for carrying out the hydrogenation in batch mode, the reaction was investigated using the FlowSYNTH reactor. The best test (Entry 4) was taken as the reference point, and the catalyst amount for all experiments run in the MW-assisted flow reactor was not changed. As a first step, we focused on the influence of hydrogen pressure and reaction temperature, while keeping slurry-solution flow, residence time (t_r_) and, consequently, run time constant. The concept of developing a quick process supported by leak tests with the slurry solution directed us towards using a short residence time. The slurry-solution flow was set at 150 mL/min, the hydrogen flow rate between 150–200 NmL/min, with 80 s of residence time (considering only the liquid phase) and each run taking 7.5 min. The system was flushed with THF and nitrogen to clean the reactor and lines after each loop.

A pressure range of 4.0 bar to 8.0 bar was investigated, with MW irradiation being applied between 30–35 °C and the starting solution being flowed for one cycle. Under these conditions, we did not observe experimental evidence for the bis-protected meropenem (**4**) deblock in meropenem (**1**). Each analysis was performed on the aqueous solution after catalyst filtration.

Since pressure and temperature were observed to have no measurable impact, we decided to increase the residence time for the next experiments and use the same pressure conditions as Entry 4 (6.8 bar). This decision was taken to give more time to the hydrogen, with the catalyst, to react with the substrate. To ensure that the reactor, and consequently the slurry, are sufficiently pulsed with MW irradiation, the temperature was set at 35 °C; in order to achieve these new conditions, the slurry-solution flow was decreased and, accordingly, the run time was increased.

With the solution flow set at 105 mL/min and the hydrogen flow at 100 NmL/min to give 115 s of residence time and 10.5 min of run time, we obtained a decrease in bis-protected meropenem (**4**) concentration in a single cycle, as indicated in Table 2. Compared to the batch experiments, an 88% value of raw materials was still high; thus, the slurry was run until the complete disappearance of the substrate. After ten cycles and a total residence time of 1150 s (19 min), we obtained a meropenem (**1**) yield in solution of 69% with a bis-protected meropenem (**4**) residual of 3%. A progressive and constant decrease in the substrate has been observed without altering the catalyst activity.

The same experiment was repeated with a solution flow of 57 mL/min and a run time of 19 min, maintaining the hydrogen flow rate at 100 NmL/min, in order to have a residence time of 210 s to carry out the synthesis; substrate hydrogenation was completed in four cycles, as indicated in Table 3. A meropenem (**1**) yield in solution of 67% was obtained with a bis-protected meropenem (**4**) residual of 3% in a total residence time of 840 s (14 min). These semi-continuous tests clearly show the considerable impact of residence time on hydrogenation, making it a key parameter and necessary for reaction success. The synthesis was now performed in loop, and the total residence time was increased to allow the reaction between hydrogen and the starting materials to achieve protecting-group deblock. However, the slurry solution flow could not be reduced further to avoid the risks of clogging problems and pressure instability.

Taking the process parameters used in the Table 3 experiments as the best conditions, the hydrogenation was carried out at a higher temperature induced by MW irradiation. A clear difference between MW-assisted flow hydrogenation and the batch-mode process emerges in the data reported in Table 4. The temperature increase from 35 °C to 45 °C improved the yield from 67% to 80%, while a 5% decrease in yield was observed at 55 °C. This trend provides useful information for future industrial application, as it clearly highlights the subtle balance between the operating parameters and the instability of the carbapenem skeleton. The combination of better mixing in the flow reactor with the uniform distribution of MW irradiation improved the yield of meropenem (1) in solution after catalyst filtration, while the opposite result was observed in experiments in the Parr reactor when the temperature was increased. A solution yield of 80% is slightly lower than the batch process data, as was the yield of the isolated product (70% vs. 74%). However, the benefits of running a semi-continuous process would enhance productivity and eliminate the small yield deviation.

**Table 4:** In-loop hydrogenation test at 45 °C and 55 °C and 6.8 bar.

Entry	Cycles	Temperature (°C)	Total Residence Time (s)	Meropenem (1) Yield in Solution (%) after Catalyst Filtration	Crude Meropenem (1) Yields (%)
23	4	45	840	80	70
24	4	55	840	75	65

Reaction conditions: The reaction conditions are reported in Materials and Methods in Section 2.1.2. The FlowSYNTH reactor is described in Materials and Methods in Section 2.2.2. The catalyst amount was 21% dry w/w with reference to bis-protected meropenem (**4**), as reported in Table 1, entry 4.

It is worth noting that the product quality obtained using MW-assisted flow hydrogenation is equivalent to that obtained using the batch process (98%). An LC-MS investigation of the crude meropenem (**1**), from Entry 23, showed the same impurities as reported in Figure 2 at the same values, confirming that they are characteristic of this specific reaction applied to this carbapenem.

## 4. Conclusions

An experimental study into the MW-assisted catalytic hydrogenation of bis-protected meropenem (**4**) has been carried out. The FlowSYNTH reactor has been used to this purpose, giving a meropenem (**1**) yield in solution after catalyst filtration of 80% with an isolated-product yield of 70%. The optimization of residence time (840 s) and cycle number (4) has led to the development of a novel protocol that has halved the reaction time (14 min vs. 30 min). Using MW irradiation, the deblock reaction was completed in only 14 min, whereas the batch process required 30 min. To achieve this result, the solution underwent four cycles with a residence time of 210 s per loop, with a solution flow of 57 mL/min at 45 °C and 6.8 bar. This technology has proven to be softer than the Parr reactor as it can achieve a similar yield result at a higher temperature. The carbapenem nucleus has been preserved and product quality is comparable to that of the batch product. Two characteristic impurities in the hydrogenation process have been found, and they are present both in the products of the MW-assisted flow process and the batch process, confirming that they are specific to this reaction. The results obtained using this technology show, once again, its potential and adaptability, even in the synthesis of an important carbapenem, such as meropenem. It is our hope that this work can pave the way for new opportunities for this emerging technology in the pharmaceutical world.

## Figures and Tables

**Table 1:** Screening of hydrogenation conditions in the Parr reactor.

Entry	H_2_ (bar)	Temperature (°C)	Catalyst ^a^ (g)	Dry Catalyst Weight/Substrate Weight (%)	Time (Min)	Meropenem (1) Yield in Solution (%) after Catalyst Filtration	Crude Meropenem (1) Yields (%)
1	20	37	5.7	10.0	30	66	-
2	20	37	2.8	5.0	60	63	-
3	20	37	1.4	2.5	120	53	-
4	6.8	30	12.0	21.0	30	89	74
5	6.8	30	6.0	10.5	60	84	71
6	6.8	35	6.0	10.5	60	75	-
7	6.8	45	6.0	10.5	60	60	-
8	6.8	30	3.0	5.3	90	71	61

Reaction conditions: The reaction conditions are reported in Materials and Methods in Section 2.1.2. The Parr reactor is described in Materials and Methods in Section 2.2.1. ^a^ The 10% Pd/C weight is to be considered 50% wet.

**Table 2:** Semi-continuous hydrogenation test with t_r_ = 115 s for one loop at 6.8 bar.

Entry	Cycle	*Bis*-Protected Meropenem (4) Residual (%)
9	1st	88
10	2nd	69
11	3rd	51
12	4th	38
13	5th	24
14	6th	16
15	7th	10
16	8th	5
17	9th	4
18	10th	3

Reaction conditions: The reaction conditions are reported in Materials and Methods in Section 2.1.2. The FlowSYNTH reactor is described in Materials and Methods in Section 2.2.2. The catalyst amount was 21% dry w/w with reference to bis-protected meropenem (**4**) as reported in Table 1, entry 4.

**Table 3:** In-loop hydrogenation test with t_r_ = 210 s for one loop and 6.8 bar.

Entry	Cycle	*Bis*-Protected Meropenem (4) Residual (%)
19	1st	66
20	2nd	38
21	3rd	11
22	4th	3

Reaction conditions: The reaction conditions are reported in Materials and Methods in Section 2.1.2. The FlowSYNTH reactor is described in Materials and Methods in Section 2.2.2. The catalyst amount was 21% dry w/w with respect to bis-protected meropenem (**4**), as reported in Table 1, entry 4.

## Data Availability

Data is contained within this article or Supplementary Materials.

## Supplementary Materials

The following supporting information can be downloaded at: https://www.mdpi.com/article/10.3390/pharmaceutics15051322/s1, Figure S1: ^1^H-NMR of bis-protected meropenem (**4**). Figure S2: ^13^C-NMR of bis-protected meropenem (**4**). Figure S3: ^1^H-NMR of meropenem (**1**). Figure S4: ^13^C-NMR of meropenem (**1**). Figure S5: LC-MS analysis of crude meropenem (**1**).
