# Supplementary material for: From Batch to the Semi-Continuous Flow Hydrogenation of pNB, pNZ-Protected Meropenem
Source: Pharmaceutics. 2023 Apr 23;15(5):1322. doi: 10.3390/pharmaceutics15051322 (PMC10224265; doi:10.3390/pharmaceutics15051322)
Supplement: Supplementary file 1 [file pharmaceutics-15-01322-s001.zip › pharmaceutics-2299468-supplementary.pdf]

Supplementary Material

From Batch to the Semi-Continuous Flow Hydrogenation of *p*NB, *p*NZ-Protected Meropenem

Marziale Comito [1,2], Riccardo Monguzzi [2], Silvia Tagliapietra [1], Angelo Maspero [3], Giovanni Palmisano [3] and Giancarlo Cravotto [1,*]

[1] Dipartimento di Scienza e Tecnologia del Farmaco, University of Turin, Via Pietro Giuria 9, 10125 Turin, Italy
[2] Research and Development, ACS Dobfar SpA, Via Paullo 9, 20067 Tribiano, Italy
[3] Dipartimento di Scienza e Alta Tecnologia, University of Insubria, Via Valleggio 9, 22100 Como, Italy;
* Correspondence: giancarlo.cravotto@unito.it

Figure captions

Figure S1: ^{1}H-NMR of bis-protected meropenem (**4**).

Figure S2. ^{13}C-NMR of bis-protected meropenem (**4**).

Figure S3. ^{1}H-NMR of meropenem (**1**).

Figure S4. ^{13}C-NMR of meropenem (**1**).

Figure S5. LC-MS analysis of crude meropenem (**1**).

Figure S1

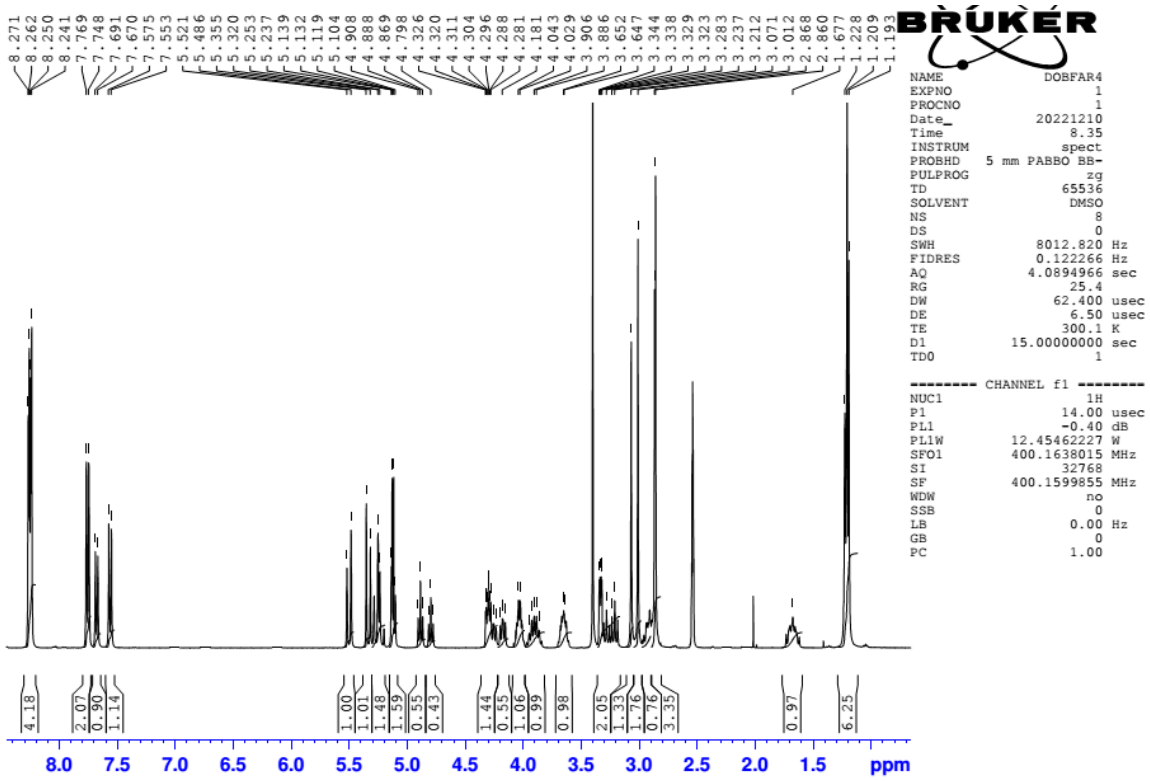
BRUKER
NAME DOBFAR4
EXPNO 1
PROCNO 1
Date_ 20221210
Time 8.35
INSTRUM spect
PROBHD 5 mm PABBO BB-
PULPROG zg
TD 65536
SOLVENT DMSO
NS 8
DS 0
SWH 8012.820 Hz
FIDRES 0.122266 Hz
AQ 4.0894966 sec
RG 25.4
DW 62.400 usec
DE 6.50 usec
TE 300.1 K
D1 15.00000000 sec
TD0 1
======== CHANNEL f1 ========
NUC1 1H
P1 14.00 usec
PL1 -0.40 dB
PL1W 12.45462227 W
SFO1 400.1638015 MHz
SI 32768
SF 400.1599855 MHz
WDW no
SSB 0
LB 0.00 Hz
GB 0
PC 1.00
ppm

Figure S2

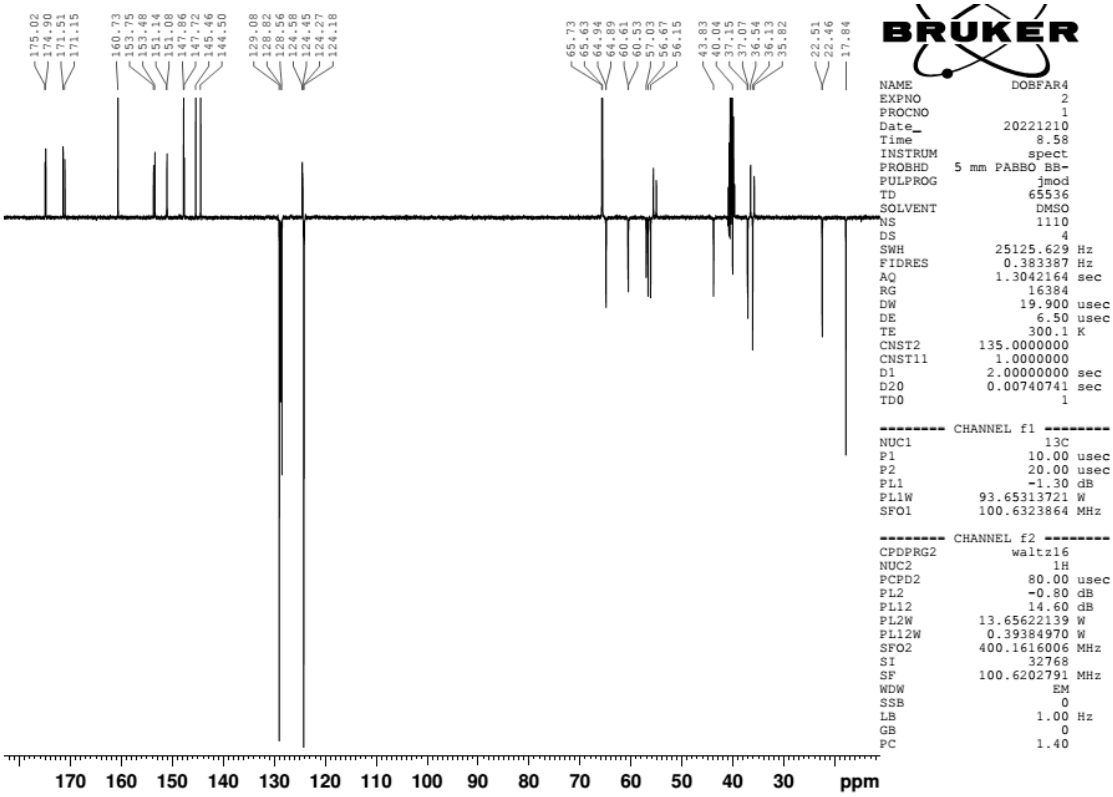
175.02
174.90
171.51
171.15
160.73
153.75
153.48
151.14
151.08
147.86
147.72
145.46
144.50
129.08
128.82
128.56
124.58
124.45
124.27
124.18
65.73
65.63
64.94
64.89
60.61
60.53
57.03
56.67
56.15
43.83
40.04
37.15
37.07
36.54
36.13
35.82
22.51
22.46
17.84
BRUKER
NAME DOBFAR4
EXPNO 2
PROCNO 1
Date_ 20221210
Time 8.58
INSTRUM spect
PROBHD 5 mm PABBO BB-
PULPROG jmod
TD 65536
SOLVENT DMSO
NS 1110
DS 4
SWH 25125.629 Hz
FIDRES 0.383387 Hz
AQ 1.3042164 sec
RG 16384
DW 19.900 usec
DE 6.50 usec
TE 300.1 K
CNST2 135.0000000
CNST11 1.0000000
D1 2.00000000 sec
D20 0.00740741 sec
TD0 1
======== CHANNEL f1 ========
NUC1 13C
P1 10.00 usec
P2 20.00 usec
PL1 -1.30 dB
PL1W 93.65313721 W
SFO1 100.6323864 MHz
======== CHANNEL f2 ========
CPDPRG2 waltz16
NUC2 1H
PCPD2 80.00 usec
PL2 -0.80 dB
PL12 14.60 dB
PL2W 13.65622139 W
PL12W 0.39384970 W
SFO2 400.1616006 MHz
SI 32768
SF 100.6202791 MHz
WDW EM
SSB 0
LB 1.00 Hz
GB 0
PC 1.40
170 160 150 140 130 120 110 100 90 80 70 60 50 40 30 ppm

Figure S3

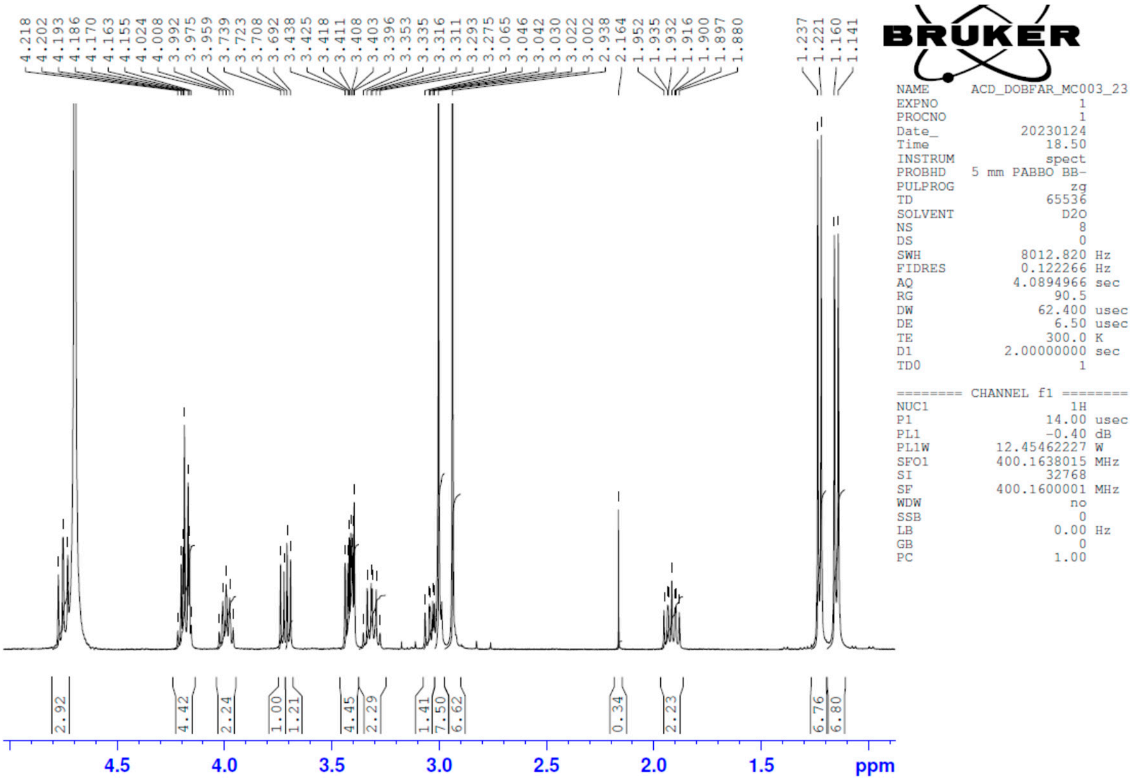
4.218
4.202
4.193
4.186
4.170
4.163
4.155
4.024
4.008
3.992
3.975
3.959
3.739
3.723
3.708
3.692
3.438
3.425
3.418
3.411
3.408
3.403
3.396
3.353
3.335
3.316
3.311
3.293
3.275
3.065
3.046
3.042
3.030
3.022
3.002
2.938
2.164
1.952
1.935
1.932
1.916
1.900
1.897
1.880
1.237
1.221
1.160
1.141
BRUKER
NAME ACD_DOBFAR_MC003_23
EXPNO 1
PROCNO 1
Date_ 20230124
Time 18.50
INSTRUM spect
PROBHD 5 mm PABBO BB-
PULPROG zg
TD 65536
SOLVENT D2O
NS 8
DS 0
SWH 8012.820 Hz
FIDRES 0.122266 Hz
AQ 4.0894966 sec
RG 90.5
DW 62.400 usec
DE 6.50 usec
TE 300.0 K
D1 2.00000000 sec
TD0 1
======== CHANNEL f1 ========
NUC1 1H
P1 14.00 usec
PL1 -0.40 dB
PL1W 12.45462227 W
SFO1 400.1638015 MHz
SI 32768
SF 400.1600001 MHz
WDW no
SSB 0
LB 0.00 Hz
GB 0
PC 1.00
2.92
4.42
2.24
1.00
1.21
4.45
2.29
1.41
7.50
6.62
0.34
2.23
6.76
6.80
4.5
4.0
3.5
3.0
2.5
2.0
1.5
ppm

Figure S4

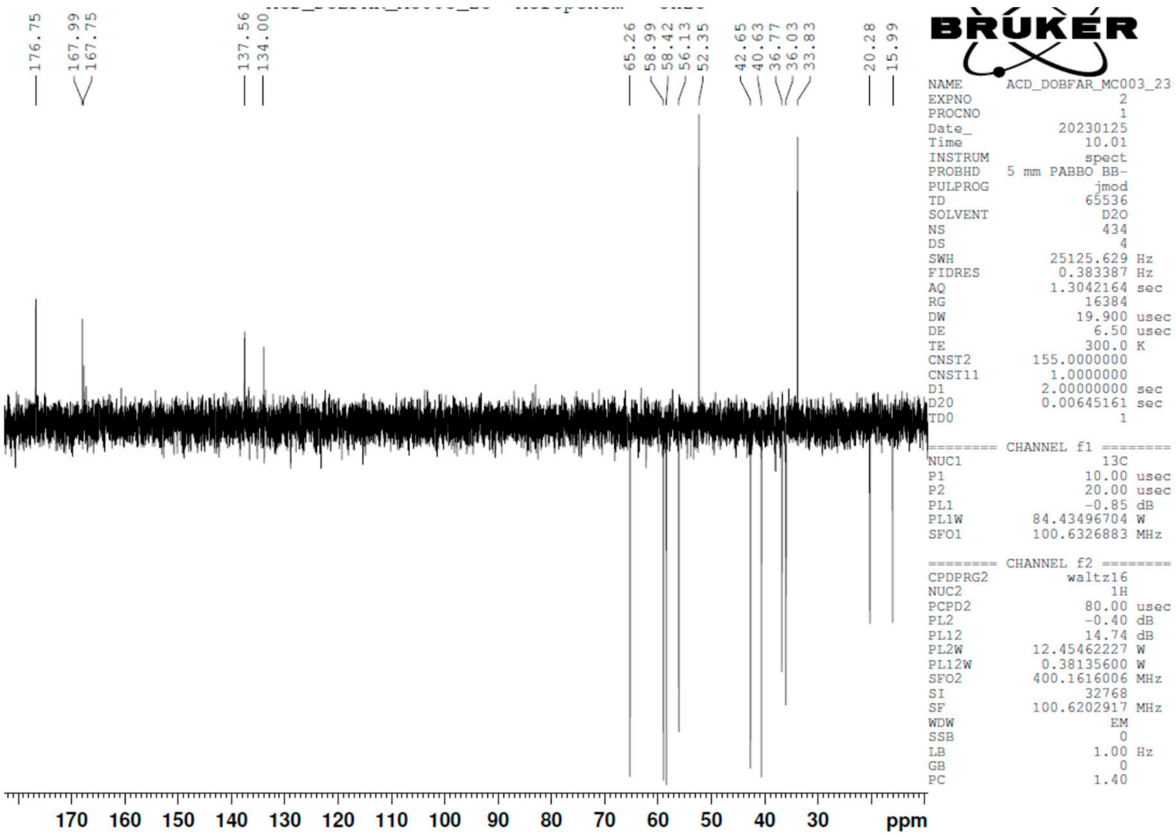

176.75
167.99
167.75
137.56
134.00
65.26
58.99
58.42
56.13
52.35
42.65
40.63
36.77
36.03
33.83
20.28
15.99
BRUKER
NAME ACD_DOBFAR_MC003_23
EXPNO 2
PROCNO 1
Date_ 20230125
Time 10.01
INSTRUM spect
PROBHD 5 mm PABBO BB-
PULPROG jmod
TD 65536
SOLVENT D2O
NS 434
DS 4
SWH 25125.629 Hz
FIDRES 0.383387 Hz
AQ 1.3042164 sec
RG 16384
DW 19.900 usec
DE 6.50 usec
TE 300.0 K
CNST2 155.0000000
CNST11 1.0000000
D1 2.00000000 sec
D20 0.00645161 sec
TD0 1
======== CHANNEL f1 ========
NUC1 13C
P1 10.00 usec
P2 20.00 usec
PL1 -0.85 dB
PL1W 84.43496704 W
SFO1 100.6326883 MHz
======== CHANNEL f2 ========
CPDPRG2 waltz16
NUC2 1H
PCPD2 80.00 usec
PL2 -0.40 dB
PL12 14.74 dB
PL2W 12.45462227 W
PL12W 0.38135600 W
SFO2 400.1616006 MHz
SI 32768
SF 100.6202917 MHz
WDW EM
SSB 0
LB 1.00 Hz
GB 0
PC 1.40
170 160 150 140 130 120 110 100 90 80 70 60 50 40 30 ppm

Figure S5

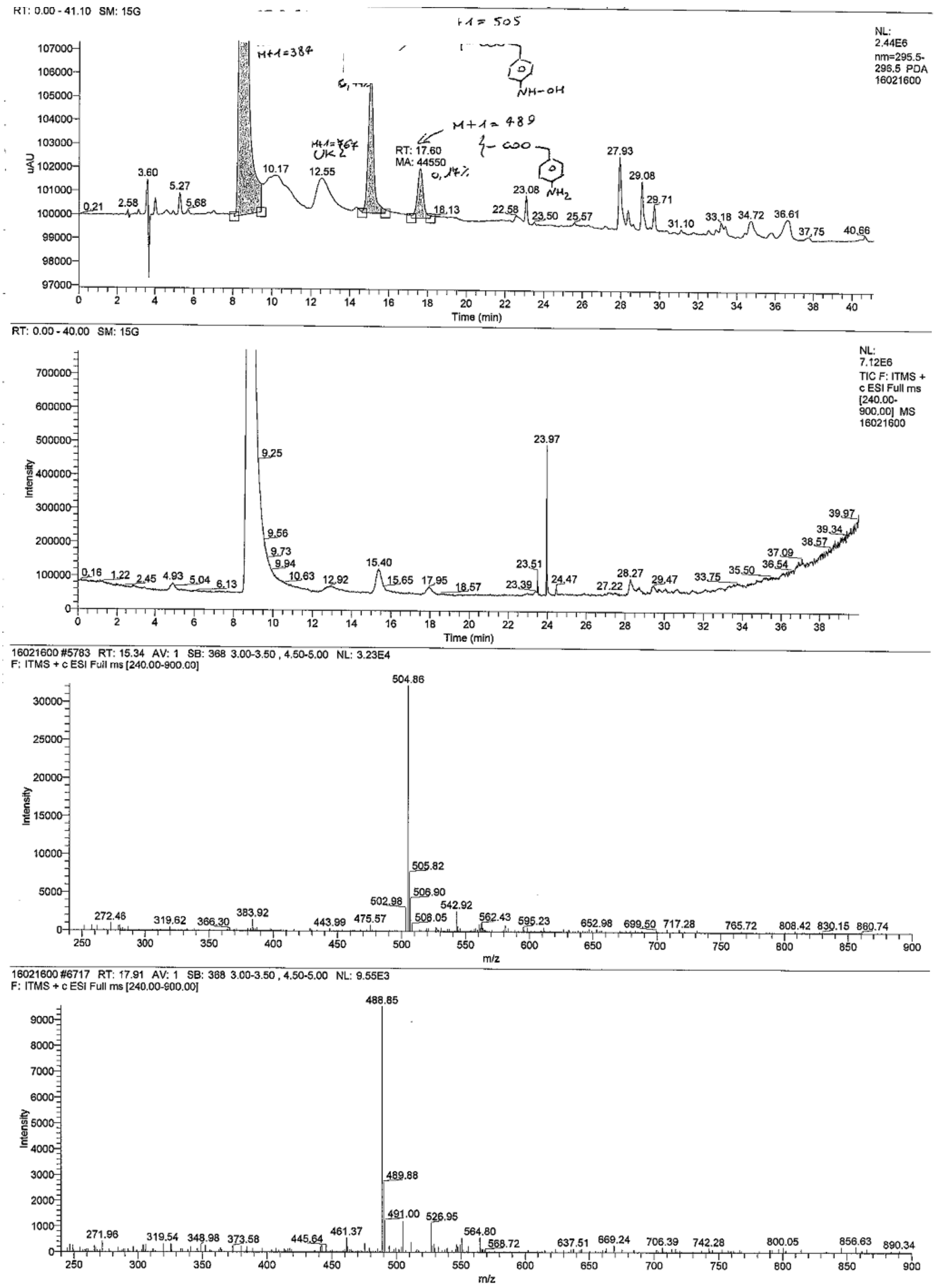
RT: 0.00 - 41.10 SM: 15G
M+1=384
+1= 505
NH-OH
M+1=767
UK 2
RT: 17.60
MA: 44550
0,14%
M+1= 489
NH2
NL: 2.44E6 nm=295.5-296.5 PDA 16021600
uAU
Time (min)
RT: 0.00 - 40.00 SM: 15G
NL: 7.12E6 TIC F: ITMS + c ESI Full ms [240.00-900.00] MS 16021600
Intensity
Time (min)
16021600 #5783 RT: 15.34 AV: 1 SB: 368 3.00-3.50 , 4.50-5.00 NL: 3.23E4
F: ITMS + c ESI Full ms [240.00-900.00]
504.86
505.82
506.90
Intensity
m/z
16021600 #6717 RT: 17.91 AV: 1 SB: 368 3.00-3.50 , 4.50-5.00 NL: 9.55E3
F: ITMS + c ESI Full ms [240.00-900.00]
488.85
489.88
491.00
Intensity
m/z
